# Cranial Nerve Affection in Adolescents with Type 1 Diabetes Assessed by Corneal Confocal Microscopy, Smell and Taste Tests

**DOI:** 10.1155/2023/2709361

**Published:** 2023-03-31

**Authors:** Vinni Faber Rasmussen, Dorthe Rasmussen, Mathilde Thrysøe, Páll Karlsson, Mette Madsen, Kurt Kristensen, Jens Randel Nyengaard, Astrid Juhl Terkelsen, Esben Thyssen Vestergaard, Therese Ovesen

**Affiliations:** ^1^Danish Pain Research Center, Department of Clinical Medicine, Aarhus University, Aarhus, Denmark; ^2^Department of Pediatrics and Adolescent Medicine, Randers Regional Hospital, Randers, Denmark; ^3^Steno Diabetes Center Aarhus, Aarhus University Hospital, Aarhus, Denmark; ^4^Department of Clinical Medicine, Aarhus University, Aarhus, Denmark; ^5^Core Centre for Molecular Morphology, Section for Stereology and Microscopy, Department of Clinical Medicine, Aarhus University, Aarhus, Denmark; ^6^Department of Pediatric and Adolescent Medicine, Aalborg University Hospital, Aalborg, Denmark; ^7^Steno Diabetes Center North, Aalborg, Denmark; ^8^Department of Pathology, Aarhus University Hospital, Aarhus, Denmark; ^9^Department of Neurology, Aarhus University Hospital, Aarhus, Denmark; ^10^University Clinic for Flavour Balance and Sleep Ear Nose and Throat Department, Gødstrup Hospital, Herning, Denmark

## Abstract

**Aim:**

To determine whether adolescents with type 1 diabetes (T1D) have morphological changes of the corneal nerve fibers and reduced smell and taste function compared to healthy control subjects as a sign of cranial nerve affection and to evaluate possible associated risk factors for cranial nerve affection.

**Methods:**

The study was a part of the T1DANES study including 60 adolescents (15–<19 years) and 23 healthy age-matched controls. First, clinical and biochemical data on the participants were obtained, and the second step involved a test day with neurological examinations including corneal confocal microscopy (CCM), olfactory testing with Sniffin' Sticks, and gustatory assessment with taste-drop test.

**Results:**

The adolescents with T1D (mean diabetes duration 9.8 years, mean HbA1c 61 mmol/mol) had lower CCM parameters (corneal nerve fiber density, corneal nerve branch density, corneal nerve fiber length, and corneal nerve fiber fractal dimension) compared to control subjects (all *p* < 0.05). No differences in total score for smell test (*p* = 0.66) and taste test (*p* = 0.47) were found, but adolescents with T1D had reduced ability to taste sweet (*p* < 0.01). In total, 24% had two or more reduced CCM parameters, 12% had reduced smell test, and 23% had abnormal taste test. Higher waist to height ratio (WHtR) was the only risk factor found for reduced corneal nerve fiber density, and higher BMI-SDS and WHtR were found for impaired taste function. Having abnormal smell test increased the risk for having abnormal taste perception, and vice versa.

**Conclusion:**

Up to 29% of adolescents with T1D had abnormal test scores indicating cranial nerve affection. Lower corneal nerve fiber density and reduced ability to taste sweet were found in adolescents with T1D compared to control subjects. Clinical attention to smell and taste function seems important because it requires intervention for advising adolescents with impaired smell and taste function.

## 1. Introduction

Previous studies have shown that 10 to 75% of adolescents with type 1 diabetes (T1D) have neuropathy [1], which is concerning, as neuropathy is known to have negative consequences on health outcome and quality of life [2]. Diabetes is a well-known risk factor for nerve damage both in the peripheral and central nervous system (CNS) [3]. The cranial nerves (CN) are mainly considered components of the peripheral nervous system, except for the olfactory (CNI) and optic (CNII) nerves which emerge from the cerebrum and, on a structural level, are more considered part of the CNS. Cranial nerves relay information between the brain and mainly the regions of the head and neck, including senses [4, 5].

Examination of the cranial nerves has been stated as an important part of a complete neurological examination, because affection of each cranial nerve can lead to various symptoms [6]. In this paper, we focus on CN affection represented by trigeminal derived branches (CNV) in the cornea, as well as olfactory (CNI) and gustatory nerves (CNVII, CNIX, and CNX).

The ophthalmic division of CNV innervates the cornea and the morphology of these nerves can be assessed by corneal confocal microscopy (CCM) [7]. CNV is involved in the corneal blink reflex and activation of tear production, which is stimulated by irritants touching the cornea. The sensory information is transmitted through CNV, whereas the facial nerve (CNVII) activates the responses [8]. Affection of the ophthalmic division of CNV can lead to impaired eye protection against foreign bodies with reduced possibility to wash irritants away with risk for eye damage and infection [8]. A proper olfactory and gustatory function is also dependent of the normal function of cranial nerves, and these functions can be evaluated by presenting different odors and flavors followed by responding to a series of response alternatives. More validated tests exist including Sniffin' Stick olfactory test and taste-drop test [9, 10].

However, despite available tests of cranial nerve functions and the high prevalence of neuropathy in adolescents with T1D, a limited number of studies have investigated cranial nerve affection in adolescents with T1D. Cozzini et al. found reduced corneal nerve fiber density in adolescents with T1D compared to healthy control subjects [11]. Yilmaz et al. found reduced smell function in 30 children with T1D compared to control subjects, but within the normal ranges [12], and Catamo et al. demonstrated impaired taste perception in young individuals (aged 6 to 20 years) with T1D [13]. The sparse number of studies makes it difficult to conclude whether cranial nerve affection in adolescents with T1D is a problem or not, and studies of adults with T1D have shown contradictory results [14–18]. Nonetheless, investigation of adolescents with T1D seems useful because of fewer comorbidities and age-related confounders compared to adults making it preferable to target adolescents in order to obtain knowledge of the diabetic impact on cranial nerves.

The primary aim of this study was to investigate whether adolescents with a T1D duration of more than 5 years have morphological changes in the corneal nerves and reduced the smell and taste function compared to healthy control subjects as signs of CN affection. The secondary aim was to identify possible risk factors for CN affection.

## 2. Materials and Methods

### 2.1. Study Population

The study is a part of the T1DANES study. Adolescents with T1D were recruited among patients attending the outpatient clinics at Danish hospitals in Randers, Aarhus and Aalborg and Steno Diabetes Centre, Aarhus and North Denmark, between August 2020 and December 2021. Inclusion criteria were age 15–<19 years and a diabetes duration >5 years. Exclusion criteria were medical treatment or other diseases that could affect the central or peripheral nervous system. Additionally, participants who had tested positive for COVID-19 within 72 hours before the test day and those with a known history of COVID-19 were excluded from the study. A healthy age-matched control group was enrolled via advertisement at a boarding school and secondary schools.

Informed oral and written consent was obtained from each participant and the accompanying parents. All procedures in the study protocol were approved by the Danish Ethics Committee (Project ID M-2019-211-19) and Legal Office, Central Denmark Region (1-16-02-42-21).

### 2.2. Clinical and Biochemical Data Collection

All participants underwent an examination day at Aarhus University Hospital, lasting from 8.00 a.m. to about 1 p.m. The participants showed up fasting, and the adolescents with T1D were taking their normal basal insulin dose. All available clinical and biochemical data were extracted from the adolescent's clinical electronic files, and in case of missing data, the supplemental data were collected on the test day. All participants had a fasting blood sample taken for later analysis, and additionally, the blood from control subjects was analyzed for HbA1c and lipid profile. Data from patients were extracted from the last visit at the hospital. The weight and height of each participant were measured, and BMI (kg/m^2^) was calculated as weight divided by the square of height. Hip and waist circumferences were obtained with a measuring tape, and blood pressure and heart rate were recorded by an automatic blood pressure monitor. The stage of puberty was obtained by self-assessment showing illustrations of the different Tanner stages. Information about current and previous infections with COVID-19, activity levels, alcohol consumption, and smoking habits was obtained by self-reporting. Quality of life was evaluated by the WHO-5well-being index [19].

### 2.3. Assessment of Cranial Nerve Affection

To assess the morphology of the small nerve fiber in the cornea, Heidelberg Retina Tomograph III laser-scanning confocal microscope (Heidelberg Engineering GmbH, Heidelberg, Germany) was used.

CCM uses fluorescence optics, and the method and diagnostic utility in patients with diabetes are previously described [20]. Both eyes of the participants were examined after topical anesthesia and viscous eye gel. From approximately 100 images collected during a bilateral CCM procedure, three images from each eye were selected in a standardized process for final automated analysis. The parameters corneal nerve fiber density (CNFD), corneal nerve fiber length (CNFL), corneal nerve branch density (CNBD), corneal nerve total branch density (CTBD), and corneal nerve fiber fractal dimension (CNFraD) were quantified using the software ACC-metrics (CC-metrics: M. A. Dabbah, Imaging Science, University of Manchester, United Kingdom). The CCM data obtained from healthy control subjects were used for defining abnormal results (<5 percentile).

The validated Sniffin' Sticks test was performed by one trained examiner to assess the olfactory function. The test included three subtests evaluating threshold (T), discrimination (D), and identification (I) giving a total TDI (threshold, discrimination, and identification) score. Odorants were presented by removing the cap from the Sniffin' Stick pens (Burghart Messtechnik, Wedel, Germany) and were presented to both nostrils, approximately three seconds per individual pen. The participants were guided through the three subtests, of which method and validation previously have been published [9]. Normative data obtained by Hald et al. were used for defining abnormal results [21] (abnormal if TDI <28), because of a bigger sample size of healthy adolescents.

To assess the gustatory function, the taste-drop test (TDT) was performed by the same trained examiner performing the Sniffin' Stick test. The flavors sour, sweet, salty, and bitter were presented to the participants with a transfer pipette (approximately 20 *μ*L) to the desired region of the tongue. A list of options was presented to the participant who had to identify the taste qualities. All four tastes were presented in semirandomized rounds with the lowest concentration first, before initiating the next round with a higher concentration. The taste sensitivity scores for all four tastes were determined according to the previously described procedure given a total TDT score [10]. Normative data obtained by Fjaeldstad et al. were used for defining abnormal results [10, 22] (abnormal if TDT <29), due to a bigger sample size of healthy adolescents.

### 2.4. Statistical Analysis

All statistical analyses were performed in the software program R (R Core Team (2022), Vienna, Austria). All variables in Table 1 were tested for normal distribution by Shapiro–Wilk test and QQ plot. Descriptive data are presented as mean (SD), median (range), and number (%) for continuous (normal distribution), continuous (skewed distribution), and categorical variables, respectively. Data between groups were compared using Student's *t*-test for continuous variables with normal distribution; Wilcoxon rank sum test for nonparametric continuous variables; and Fisher's exact test for categorical variables. *p* values <0.05 were considered significant for all data analyses. For calculating relative risk ratios, the function *epi.2by2* in R was used, which measures the risk, and a chi-squared test for difference in the observed proportion from count data is presented in a 2 by 2 table.

## 3. Results

**Figure 1:**
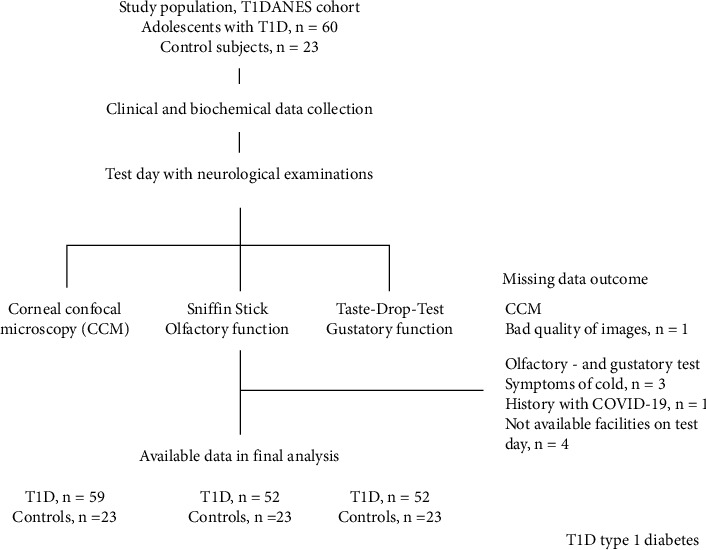
Flowchart of the study population selection and number of tests in the final analysis.

In total, 60 adolescents with T1D and 23 control subjects were enrolled.  Figure 1 shows the study population selection and the number of tests available for analysis, and Table 1 shows the characteristics of the participants. One participant was excluded due to a known COVID-19 infection four months before the test day.

### 3.1. Corneal Nerve Morphology and Olfactory and Gustatory Function

**Figure 2:**
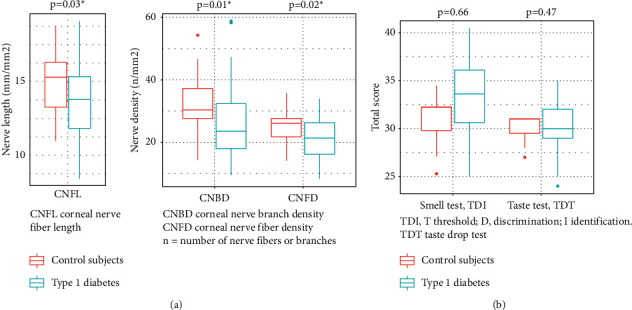
Results of quantification of the corneal nerves (a) and results of the smell and taste test (b).

Table 2 shows the results of CCM, and CNFD, CNBD, CNFL, and CNFraD were significantly lower in adolescents with T1D compared to the control subjects.  Figure 2 shows the data presented in boxplots of CNFL, CNFD, and CNBD, and the data of total scores from the smell and taste tests.

No differences in total scores of the smell and taste test were found when comparing adolescents with T1D and control subjects: TDI score mean 33.3 (SD 3.8) vs. 29.7 (SD 2.5), *p*=0.66; TDT score mean 29.7 (SD 2.6) vs. 28.9 (SD 1.2), *p*=0.47.

Evaluation of the different subparts of the smell test showed that adolescents with T1D had significantly higher mean in threshold (T) and identification (I), but similar discrimination (D) compared to control subjects (T score 6.4 (SD 2.4) vs. 5.1 (SD 1.4), *p*=0.03; D score 12.3 (SD 1.9) vs. 10.8 (SD 1.5), *p*=0.66 I score. 14.6 (SD 1.2) vs. 13.8 (SD 1.3), *p*=0.04).

In the taste-drop test, the adolescents with T1D had reduced perception to taste sweet compared to control subjects (sweet score 6.8 (SD 1.1) vs. 7.3 (SD 0.8), *p* < 0.01). No differences in the other flavors were found (sour score 6.9 (SD 0.8) vs. 6.4 (SD 0.6), *p*=0.21; salty score 8.1 (SD 0.8) vs 7.6 (SD 0.5), *p*=0.30; bitter score 7.9 (SD 1.4) vs 7.7 (0.4), *p*=0.74).

### 3.2. Prevalence of Cases with Abnormal Results on CCM and Smell and Taste Tests

In total, 29% (17/59) of the adolescents with T1D had reduced CNFD, 17% (10/59) had reduced CNBD, 8% (5/59) had reduced CNFL, and 14% (8/59) had reduced CNFraD, when using <5 percentile of data obtained from healthy control subjects as cut off. In total, 12% (7/59) had only one reduced CCM parameter, and 24% (14/59) adolescents had a minimum of two reduced CCM parameters. Six adolescents (10%) had reduced CNFD, CNBD, and CNFL.

In addition, 12% (6/52) of the adolescents with T1D had reduced smell function, and 23% (12/52) had reduced taste function using cut-off from normative data obtained by Hald et al. [21] and Fjaeldstad et al. [10].

### 3.3. Risk Factors for Abnormal Test

Table 3 shows the clinical and biochemical variables and the relative risk ratio (RR) for abnormal tests of CCM and smell and taste tests. Increased waist circumference to height ratio was a significant risk factor for reduced CNFD (RR 2.31 (1.08, 2.35)) and impaired taste function (RR 3.21 (1.31, 7.89)).

Having an abnormal smell test increased the risk of having an abnormal taste test (RR 3.83 (1.64, 8.94)), and vice versa (RR 6.67 (1.39, 32.04). Reduced CNFD was not associated with an increased relative risk of abnormal smell test (RR 0.59 (0.15, 2.32)) or abnormal taste test (RR 0.58 (0.15, 2.33)).

### 3.4. Abnormal Test and Impact on Quality of Life

Abnormal CNFD or reduced smell function had no significant impact on mean WHO-5well-being index in adolescents with T1D (T1D-CCM WHO-5 score 57.2 (SD 27.0) vs. 61.4 (SD 16.5), *p*=0.55; T1D-TDI WHO-5 score 46.7 (SD 22.3) vs. 62.2 (SD 17.9), *p*=0.15). The mean WHO-5well-being score in adolescents with T1D and impaired taste function vs. adolescents with T1D and normal taste function were similar (T1D-TDT WHO-5 score 60.7 (SD 16.7) vs. 60.5 (SD 20.6), *p*=0.98).

## 4. Discussion

This study aimed to explore the occurrence of signs indicating the cranial nerve affection in adolescents with T1D. We found indications of cranial nerve affection in some of the adolescents, as 24% had two reduced CCM parameters compared to controls, 12% (6/52) had reduced smell function, and 23% (12/52) had reduced taste function compared to normative data. In addition, an overall reduction of corneal nerve fiber length and densities, a better smell function, and a lower perception of the taste sweet were observed in the diabetic group compared to the control group.

Our findings of reduced CNFD, CNBD, and CNFL are in line with the findings in an Italian study investigating 150 children and adolescents (age range 10–22 years) with T1D (diabetes duration >2 years) [11]. The similarities were seen in both the exact values and the affected CCM parameters. Notably, automatic analysis has been shown to substantially underestimate CNFL compared to manual analysis [23], which could explain why our CNFL values were lower than those found by Ferdouise et al. in children with T1D and control subjects [24]. However, the overall results suggest that diabetes may lead to morphological changes in the small nerve fibers in the cornea already at a young age. How these morphological changes in cornea correlate to the structure of small nerve fibers in other body locations have been investigated in studies. A large study found that CNFL had a sensitivity of 77% for detecting sensorimotor neuropathy in 432 adults and 84 adolescents [25]. However, previous studies on diabetic populations have not reached the same consistency neither in results nor in longitudinally studies [26–28]. Accordingly, reduced CNFD is not recommended as the only marker for general peripheral small fiber neuropathy based on the current evidence [7].

The cornea nerves are the afferent component in the corneal reflex [29], and studies quantifying relationships of structural nerve changes with functional outcomes will be necessary for further understanding of what consequences reduced CNFD has for adolescents with T1D.

Knowledge about whether results suggestive of neuropathy in one body location based on one functional or structural test can indicate more generalized neuropathy is still lacking. To our knowledge, the evidence is limited and inconsistent, also in studies examining the association between smell and taste function and diabetic peripheral neuropathy [30, 31]. The use of cranial nerve tests as markers for peripheral neuropathy remains controversial, regardless of which test is selected. The fact that similar olfactory and gustatory function has been found in people with and without diabetic peripheral neuropathy has led to the idea of central neuropathy [31].

The olfactory nerve (CNI) is the only cranial nerve that projects directly to the forebrain. As mentioned, CNI is seen as a part of CNS because of its origin, and a possible link between the sense of smell, the brain, and diabetes seems possible. Insulin is known to cross the blood-brain barrier, and the olfactory bulbs are known to have the fastest transport rate of insulin into the brain [32]. This supports that blood glucose regulation with insulin may be linked to smell function. We found an overall improved smell function in adolescents with T1D compared to healthy controls. Whether this is a compensatory mechanism for their reduced ability to taste sweet or could be caused by the constant supply of basal insulin remains unclear and warrants further research. However, despite an overall improved smell function, six adolescents with T1D fulfilled the criteria for reduced smell function in our study, which supports that hyposmia is more prevalent among the patients with T1DM in our study compared with nondiabetic controls [33]. An overall impaired smell function is also observed in adults [14, 15], and altogether, further research is needed to clarify the link between diabetes and smell function. In our study, we did not find any significant risk factors when testing clinical and biochemical variables related to diabetes. The relation between olfactory dysfunction and reduced metabolic control, diabetes duration, and lipid profile remains to be unraveled in future research.

A larger epidemiological study found that the impaired smell function in adults with diabetes had a negative effect on daily food intake [14]. Association between overall taste perception and personal health parameters in younger individuals with T1D has also been described [13], and the correlation to nutrition seems important for avoiding obesity. Waist circumference to height ratio has been described as an indicator for detecting obesity in children and adolescents [34], and in our study, waist circumference to height ratio was found as a significant risk factor for impaired taste function. Initiating lifestyle changes in children and adolescents (6–18 years) with obesity has been shown to improve the gustatory function [35], it may be recommended to patients with impaired taste function. Additionally, smoking cessation may be recommended, because smoking is known to lead to olfactory loss [36]. In adults, former smoking is not a risk factor [36], and we did the same observation. However, *former* may be suspected to include a shorter period for regression in adolescents, and former smoking still requires attention.

Interestingly, we found that adolescents with T1D had an overall reduced ability to taste sweet compared to control subjects. A possible explanation could be that adolescents with T1D more often eat and drink sweet fast-acting carbohydrates for correcting low blood glucose levels, leading to more frequent sweet signals to the brain causing lesser sensitivity to the sweet flavor. A positive association between taste threshold and food preferences has been observed among adolescents [21], why further research in this area seems interesting for general health outcomes and well-being in adolescents with T1D.

It is noteworthy that the physiological changes that occur during puberty can also impact the management and development of complications associated with diabetes [37].

Limitations of our study include the fact that factors other than affected cranial nerves can impact the test results. The CCM included a risk for image selection bias, which was tried to be kept to a minimum by blinding the images with ID numbers and the choice of autonomic analysis. Confounders on all levels from chemistry to peripheral sensation to perception and brain processing [38], in addition to external factors such as early COVID-19 [39], can influence the results of the smell and taste test. In our study, risk for unknown COVID-19 infection exists; however, we did exclude all participants with symptoms and a history of former COVID-19 infection with smell and taste impairment. The T1D group also had higher alcohol consumption, which could be a confounder. Furthermore, the absence of corneal sensitivity assessment is a limitation in that it would have allowed us a more direct comparison with the results of the smell and taste test. Additionally, our study lacked comparable normative data for CCM on a larger sample size than our relatively small control group. It is also worth mentioning our comparison with already published normative data for the total smell and taste score, as we found no difference between our adolescents with diabetes and controls at the group level. However, we observed abnormal smell and taste functions among the participants when using the published data used in clinical settings.

The strength of our study was that all tests were performed in a standardized manner by the same trained healthcare professionals. The tests included in this study require equipment and trained healthcare professionals. However, these tests are commonly used in clinical settings in Denmark and are available at many hospitals.

In conclusion, we found indications of cranial nerve affection in adolescents with type 1 diabetes. How the reduced CCM parameters are linked to nerve dysfunction is still unknown and warrants further research. Furthermore, clinical awareness of affected smell and taste function in adolescents with T1D seems relevant due to the impact on food preferences, health parameters, and well-being. Identifying risk factors and creating intervention studies are needed for a better understanding of how to detect, treat, and prevent further progression of cranial nerve affection.

## Figures and Tables

**Table 1:** Characteristics of the study population.

Variable	Control, *N* = 23^1^	Diabetes, *N* = 60^1^	*p* value^2^
Gender (female)	16 (70%)	30 (50%)	0.14
Age (years)	16.60 (15.40–18.20)	16.90 (15.00–18.90)	0.45
HbA1c (mmol/mol)	33 (27–40)	60 (41–93)	**<0.01**
BMI (kg/m^2^)	21.12 (16.90–30.40)	22.74 (17.63–29.61)	**0.03**
BMI-SDS	0.03 (−1.80–1.68)	0.57 (−2.30–1.85)	**<0.01**
Height (cm)	174 (158–188)	173 (150–191)	0.97
Hip circumference (cm)	98 (65–112)	98 (76–114)	0.47
Waist circumference (cm)	74 (59–92)	75 (53–100)	0.49
Tanner (stage)			0.43
4	5 (22%)	19 (32%)	
5	18 (78%)	41 (68%)	
SBP (mmHg)	114 (98–130)	118 (68–147)	0.22
DBP (mmHg)	71 (59–89)	77 (55–96)	**0.03**
Pulse (BPM)	70 (55–99)	77 (48–106)	0.21
Retinopathy (yes)	0 (NA%)	3 (5.0%)	1.00
Nephropathy (yes)	0 (NA%)	2 (3.3%)	1.00
Cholesterol (mmol/L)	3.80 (2.80–5.10)	4.10 (3.00–6.40)	0.15
LDL (mmol/L)	2.10 (1.40–3.50)	2.10 (0.50–4.10)	0.90
HDL (mmol/L)	1.30 (0.68–2.20)	1.50 (0.97–3.70)	**0.04**
Triglycerides (mmol/L)	0.70 (0.30–1.10)	0.90 (0.30–3.80)	**0.01**
Alcohol (units/week)			**0.02**
0	1 (4.3%)	6 (10%)	
1–3	20 (87%)	28 (47%)	
4–7	2 (8.7%)	17 (28%)	
8–14	0 (0%)	5 (8.3%)	
>15	0 (0%)	4 (6.7%)	
Smoking (status)			0.63
Never	18 (78%)	47 (78%)	
Previous	4 (17%)	6 (10%)	
Smoke	1 (4.3%)	6 (10%)	
NI	0 (0%)	1 (1.7%)	
Activity (hours/week)			0.17
0	0 (0%)	5 (8.3%)	
1–3	2 (8.7%)	14 (23%)	
4–7	8 (35%)	19 (32%)	
>7	13 (57%)	22 (37%)	
Diabetes duration (years)	NA	8.5 (4.6–17.4)	
HbA1c mean 5 years (mmol/mol)	NA	59 (40–95)	
Total daily insulin (units/kg/day)	NA	0.85 (0.40–1.65)	
Basal insulin (units/kg/day)	NA	0.39 (0.14–0.87)	
Basal/total daily insulin ratio	NA	0.46 (0.24–0.72)	
Time in range^*∗*^ (%)	NA	55 (23–85)	
Time in hypoglycemia (%)	NA	5.0 (0.0–15.0)	

^1^Median (range) for continuous; *n* (%) for categorical. ^2^Categorical variables, fisher's exact test; continuous variable with normal-distribution, Welch two sample *t*-test; continuous variable with nonnormal distribution, Wilcoxon rank sum test. BMI, body mass index; SBP, systolic blood pressure; DBP, diastolic blood pressure; HDL, high density lipoproteins; LDL, low-density lipoproteins; *n* = number; NA, not available, NI, not indicated; yrs, years. ^*∗*^Only available data of 44 adolescents. A *p*-value of less than 0.05 was considered statistically significant.

**Table 2:** Corneal confocal microscopy parameters obtained in adolescents with type 1 diabetes and healthy controls.

	T1D	Controls	*p* value
CNFD (n/mm^2^)	21.72	25.22	0.017
CNBD (n/mm^2^)	25.59	31.59	0.013
CNFL (mm/mm^2^)	13.64	18.86	0.030
CNFraD	1.476	1.492	0.008
CTBD (n/mm^2^)	41.82	46.06	0.214

CNBD, corneal nerve branch density; CNFD, corneal nerve fiber density; CNFL, corneal nerve fiber length; CNFraD, nerve fiber fractal dimension; CTBD, corneal nerve total branch density; *N* = number of fibers or branches; T1D, type 1 diabetes.

**Table 3:** Clinical and biochemical variables and relative risk ratios for abnormal nerve test.

Variable (*n* = number with abnormal test/total number)	Relative risk ratio (confidence interval 95%)		
	Cornea nerve fiber density (*n* = 17/59)	Smell test (*n* = 6/52)	Taste test (*n* = 12/52)
HbA1c, current (mmol/mol (%))			
>75 [9] (*n* = 7)	1.14 (0.25, 5.26)	3.00 (0.26, 34.57)	0.92 (0.13, 6.46)
53–70 [7–9] (*n* = 41)	1.20 (0.30, 5.60)	0.90 (0.11, 7.12)	0.79 (0.25, 2.49)
<53 [7] (*n* = 12)	1.00	1.00	1.00
HbA1c, mean of all values last 5 yrs (mmol/mol (%))			
>75 [9] (*n* = 5)	1.47 (0.35, 6.21)	2.50 (0.39, 16.05)	1.25 (0.26, 6.07)
53–70 [7–9] (*n* = 42)	1.07 (0.37, 3.15)	0.39 (0.08, 2.05)	0.46 (0.17, 1.27)
<53 [7] (*n* = 11)	1.00	1.00	1.00
Diabetes duration (yrs)			
>10 (*n* = 17)	0.54 (0.18, 1.65)	1.23 (0.25, 6.03)	2.22 (0.85, 5.79)
5–10 (*n* = 43)	1.00	1.00	1.00
Age (yrs)			
17 to <19 (*n* = 17)	1.88 (0.87, 4.09)	NaN	1.11(0.26, 4.81)
15 to <17 (*n* = 43)	1.00	1.00	
Gender			
Male (*n* = 30)	0.86(0.38, 1.92)	1.56 (0.28, 8.56)	1.30 (0.47, 3.56)
Female (*n* = 30)	1.00	1.00	1.00
Time in range (%)			
<50 (*n* = 19)	0.79 (0.27, 2.31)	0.42 (0.05, 3.73)	0.30 (0.07, 1.21)
≥50–70 (*n* = 25)	1.00	1.00	1.00
Total insulin dose per weight per day (IE/kg/day)			
>1 (*n* = 13)	1.47 (0.64, 2.54)	0.75 (0.10, 5.74)	1.24 (0.40, 3.82)
≤1 (*n* = 46)	1.00	1.00	1.00
Basal insulin dose per weight per day (IE/kg/day)			
≥0.5 (*n* = 15)	0.63 (0.21, 1.89)	0.96 (0.13, 7.23)	0.34 (0.05, 2.35)
<0.5 (*n* = 44)	1.00	1.00	1.00
Basal/total insulin dose ratio			
>0.5 IE (*n* = 23)	1.20 (0.54, 2.68)	1.89 (0.42, 8.42)	0.94 (0.33, 2.71)
≤0.5 IE (*n* = 37)	1.00	1.00	1.00
Other microvascular complications			
Yes (*n* = 5)	0.68 (0.11, 4.09)	NaN	NaN
No (*n* = 55)	1.00	1.00	1.00
Body mass index SDS			
>1 (*n* = 15)	1.22 (0.52, 2.90)	2.71 (0.62, 11.90)	**2.71 (1.05, 7.03)**
≤1 (*n* = 44)	1.00	1.00	1.00
Waist to height ratio			
≥0.5	**2.31 (1.08, 2.35)**	3.33 (0.77, 14.42)	**3.21 (1.31, 7.89)**
<0.5	1.00	1.00	1.00
Cholesterol (mmol/l)			
≥5 (*n* = 12)	0.80 (0.28, 2.35)	2.00 (0.42, 9.42)	1.33 (0.44, 4.04)
<5 (*n* = 46)	1.00	1.00	1.00
LDL (mmol/l)			
>3 (*n* = 10)	0.63 (0.17, 2.32)	2.28 (0.49, 10.59)	1.52 (0.51, 4.51)
≤3 (*n* = 48)	1.00	1.00	1.00
Triglycerides (mmol/l)			
≥2 (*n* = 3)	2.40 (0.97, 5.95)	NaN	NaN
<2 (*n* = 55)	1.00	1.00	1.00
Smoking status			
Smoke (*n* = 6)	0.60 (0.09, 3.82)	2.20 (0.30, 16.04)	NaN
Previous (*n* = 6)	1.81 (0.72,4.55)	3.67 (0.57, 23.39)	1.33 (0.12, 18.19)
Never (*n* = 48)	1.00	1.00	1.00
Alcohol (units/week)			
≥8 (*n* = 9)	0.42 (0.06, 2.86)	NaN	0.58 (0.07, 4.72)
4–7 (*n* = 17)	1.20 (0.52, 2.75)	NaN	0.80 (0.25, 2.58)
≤3 (*n* = 34)	1.00	1.00	1.00
Activity (hrs/week)			
≤3 (*n* = 19)	1.47 (0.53, 4.03))	1.00	1.25 (0.40, 3.91)
4–7 (*n* = 19)	1.39 (0.50, 3.84)	NaN	0.84 (0.22, 3.21)
≥8 (*n* = 22)	1.00	1.00	1.00

^*∗*^NaN, not a number. None with abnormal smell test had an age between 17 and <19 years, microvascular complications, triglycerides ≥2, alcohol consumption at 4 units or more per week, and an activity level between 4 and 7 hrs./week. None with abnormal taste test had microvascular complications and triglycerides ≥2 and were smoking. Bold values = significant increased relative risk score. If the relative risk ratio >1, the confidence interval does not include 1.

## Data Availability

The datasets generated during and/or analyzed during the current study are not publicly available due to the General Data Protection Regulation but are available in an anonymized version from the corresponding author upon reasonable request and acceptance from the Legal Office, Central Denmark Region.
